# Genes Linked to Production of Secondary Metabolites in *Talaromyces atroroseus* Revealed Using CRISPR-Cas9

**DOI:** 10.1371/journal.pone.0169712

**Published:** 2017-01-05

**Authors:** Maria Lund Nielsen, Thomas Isbrandt, Kasper Bøwig Rasmussen, Ulf Thrane, Jakob Blæsbjerg Hoof, Thomas Ostenfeld Larsen, Uffe Hasbro Mortensen

**Affiliations:** Department of Biotechnology and Biomedicine, Technical University of Denmark, Søltofts Plads, Kongens Lyngby, Denmark; University of Nebraska-Lincoln, UNITED STATES

## Abstract

The full potential of fungal secondary metabolism has until recently been impeded by the lack of universal genetic tools for most species. However, the emergence of several CRISPR-Cas9-based genome editing systems adapted for several genera of filamentous fungi have now opened the doors for future efforts in discovery of novel natural products and elucidation and engineering of their biosynthetic pathways in fungi where no genetic tools are in place. So far, most studies have focused on demonstrating the performance of CRISPR-Cas9 in various fungal model species, and recently we presented a versatile CRISPR-Cas9 system that can be successfully applied in several diverse *Aspergillus* species. Here we take it one step further and show that our system can be used also in a phylogenetically distinct and largely unexplored species from the genus of *Talaromyces*. Specifically, we exploit CRISPR-Cas9-based genome editing to identify a new gene in *T*. *atroroseus* responsible for production of polyketide-nonribosomal peptide hybrid products, hence, linking fungal secondary metabolites to their genetic origin in a species where no genetic engineering has previously been performed.

## Introduction

Filamentous fungi are known as prolific producers of numerous industrially important enzymes as well as a diverse spectrum of natural products. The latter constitutes an immense reservoir of compounds of biological and medical interest, and many products originating from fungal secondary metabolism are used today in the pharmaceutical industry as *e*.*g*. antibiotics, anticancer drugs, cholesterol-lowering agents, and immunosuppressive drugs [1]. In addition, some natural products are used commercially as pigments in cosmetics, textiles, paints, and as natural food colorants [2].

The lack of genetic tools available for most fungal species has for many years been the major obstacle for exploring the molecular biology and biochemistry of all but a few model fungi. The enormous increase in sequencing projects over the past years has revealed the existence of an abundance of uncharacterized and often silent secondary metabolite gene clusters that still awaits investigation [3]. Genetic engineering of the largely unexplored fungal species would thus allow the full study of such gene clusters and could lead to the discovery and characterization of new bioactive compounds.

CRISPR-Cas9-based genetic engineering has recently been implemented in *Aspergillus nidulans* by us [4], and by others in several other species of filamentous fungi such as *Trichoderma reesei* [5], *Neurospora crassa* [6], *Magnaporta oryzae* [7], and *Penicillium chrysogenum* [8]. Our system for *A*. *nidulans* is based on an AMA1 based vector carrying genes encoding Cas9 and the sgRNA necessary for guiding the Cas9 endonuclease to the desired target sequence and can potentially be used in many fungi with little or no adaptation. In fact, the versatility of this system is demonstrated by the fact that RNA guided mutation was achieved in six different *Aspergillus* species of which one was genetically engineered for the first time [4]. Our CRISPR-Cas9 system may therefore be functional in a wide array of filamentous fungi.

**Fig 1:**
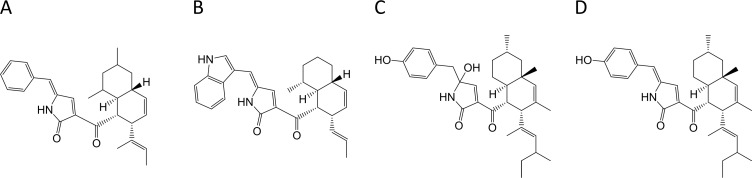
Four structurally similar polyketide-nonribosomal peptide products. Niduclavin (A) and niduporthin (B) are two novel hybrid products produced by heterologous expression of two related PKS-NRPSs in *Aspergillus nidulans* [9], while ZG-1494α/talaroconvolutin B (C) and talaroconvolutin A (D) are produced in *Talaromyces atroroseus*.

In a recent publication we reported a case of synthetic biology using *A*. *nidulans* as a host for heterologous gene expression [9]. In this study we successfully exchanged PKS- and NRPS modules between two related PKS-NRPS hybrids to produce the predicted combinations of backbone polyketide-nonribosomal peptide (PK-NRP) products [9]. However, as a surprise the synthetic polyketide-nonribosomal products contained a decalin ring system in the polyketide moiety as well as an unexpected double bond in the amino acid residue side chain, instead of the expected classical cytochalasin structure (Fig 1A and 1B). The structures of these novel derivatives are very similar to ZG-1494α (Fig 1C), an inhibitor of platelet-activating factor acetyltransferase isolated from two species of *Talaromyces*, that is *T*. *convolutes* and *T*. *atroroseus* [10,11].

For future synthetic biology efforts, we were therefore interested in identifying the genes and enzymes that are required for production of this scaffold. In *A*. *nidulans*, we speculate that the unexpected structural features in the synthetic PK-NRPs are a consequence of cross-chemical reactions catalyzed by unknown endogenous enzymes provided by the host. Hence, identification of the genes in *A*. *nidulans* is not straight forward as the origin of the chemistry is unclear. *T*. *atroroseus*, which besides ZG-1494α also produces its stereoisomer talaroconvolutin A, and the analogue talaroconvolutin B (Fig 1D), has recently been sequenced [12]. Despite that no genetic tools were available for this species prior to our work, we took advantage of this sequence and set out to identify the genetic basis of ZG-1494α and the related compound talaroconvolutin A in *T*. *atroroseus* using a bioinformatics approach and our fungal CRISPR-Cas9 technology.

## Results and Discussion

First we tested whether the CRISPR-Cas9 system that we have previously developed for *Aspergillus* could be used directly in *T*. *atroroseus*. As a simple test case for Cas9 mediated gene targeting we decided to delete the gene responsible for the green conidia pigment in *T*. *atroroseus*, which we hypothesized was formed from naphtha-γ-pyrone. To identify this gene we blasted the *A*. *nidulans*- and *A*. *niger* naphtha-γ-pyrone synthase genes (*wA* and *albA*, respectively) against the genome sequence of *T*. *atroroseus*. Amongst the homologous sequences identified in this manner, UA08_00425 was the best match as judged by the size of the ORF and by the high sequence similarities to the corresponding enzymes encoded by *wA* from *A*. *nidulans* (ID: 62%; 99% query coverage) and *albA* from *A*. *niger* (ID: 63%; 99% query coverage). UA08_00425 was therefore selected for deletion.

**Fig 2:**
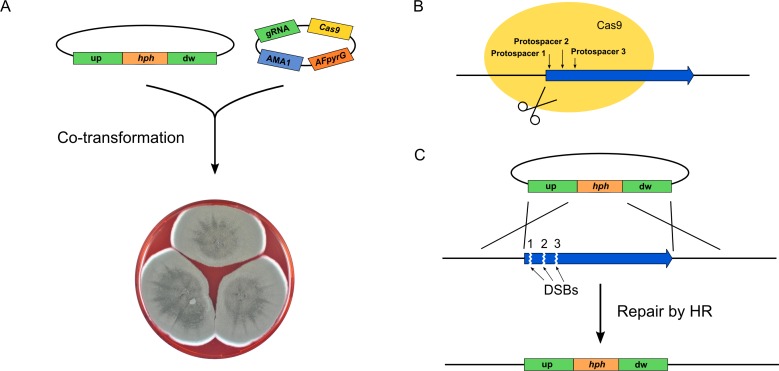
Strategy for CRISPR-Cas9 mediated deletions in *Talaromyces atroroseus*. A) *T*. *atroroseus* protoplasts were co-transformed with a circular gene-targeting substrate and an AMA1 based CRISPR-Cas9 vector containing Cas9- and sgRNA encoding genes. The transformed protoplasts were plated on medium containing hygromycin, hence, selecting for the gene-targeting substrate only. B) Three different protospacers were individually used to target the Cas9 endonuclease to UA08_00425. The positions are indicated by small vertical arrows. C) Depending on the protospacer contained by Cas9, a specific DNA DSB was produced at either position 1, 2, or 3. Repair of any of these specific DNA DSBs by homologous recombination using the circular gene-targeting substrate as repair template mediate replacement of UA08_00425 with *hph*.

Using our genetic tool box for fungal CRISPR-Cas9 gene editing [4], we constructed a plasmid containing a *hph* selection marker-based gene-targeting substrate designed for deleting UA08_00425, see Fig 2A. Since the efficiency of different protospacers are known to vary substantially [13], three AMA1 based CRISPR-Cas9 vectors encoding Cas9 and one of three different UA08_00425 specific sgRNAs were also constructed (Fig 2A and 2B).

Unlike classical gene targeting where the ends of linear gene-targeting substrates stimulate integration into the target site as they attract the homologous recombination (HR) repair machinery [14]; we exploit that a specific Cas9 induced DNA double strand break (DSB) at the target locus attracts the HR machinery [4]. As a consequence, efficient gene targeting can be achieved by using circular gene-targeting substrates as template for repair of this DNA DSB, hence, minimizing undesirable random integrations mediated by the non-homologous end-joining pathway. Using this strategy for deleting UA08_00425 (Fig 2A and 2C), we investigated the Cas9 mediated gene targeting efficiency in *T*. *atroroseus* by co-transforming the circular vector containing the UA08_00425 gene deletion sequence with each of the three UA08_00425 specific CRISPR-Cas9 vectors. AMA1 based plasmids are readily lost in the absence of selection pressure [15] and this is also the case for our AMA1 based CRISPR plasmids (see S1 Fig). To reduce the risk of undesired off-target effects, we therefore selected for the gene-targeting substrate only, and not for the *cas9* expressing AMA1 based plasmid, hence, confining *cas9* expression to the early stages of colony development.

**Fig 3:**
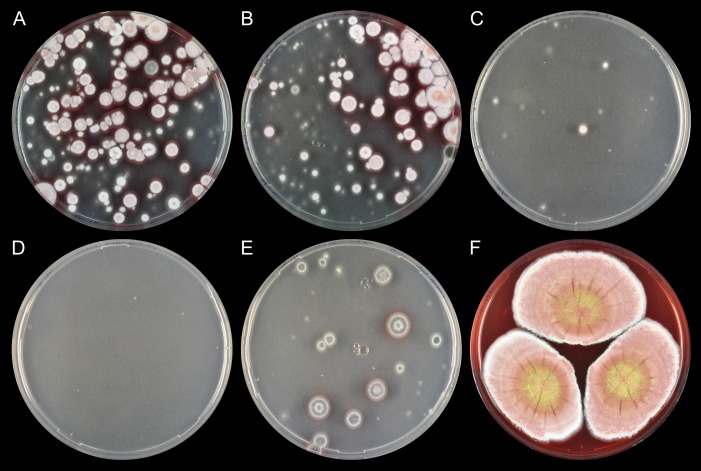
Deletion of the green pigment gene UA08_00425 in *Talaromyces atroroseus* using CRISPR-Cas9. A-C) Plates resulting from co-transformation of pD-hyg-UA08_00425 and CRISPR-Cas9 vectors carrying three different protospacers, protospacer 1–3, respectively. D) *T*. *atroroseus* transformed with gene-targeting plasmid pD-hyg-UA08_00425 (pFC574) in the absence of a CRISPR-Cas9 vector. E) *T*. *atroroseus* transformed with an AMA1-based plasmid containing the hygromycin resistance marker *hph*. F) Three-point inoculation of UA08_00425Δ *T*. *atroroseus* growing on CYA.

In two independent trials, two of the three co-transformation experiments generated numerous colonies on solid selective medium after approximately one week, whereas the remaining co-transformation produced only a few colonies (Fig 3A–3C and S2 Fig). Importantly, on all three transformation plates, close to all colonies formed white conidia spores in agreement with UA08_00425 encoding the naphtha-γ-pyrone PKS. In contrast, in the absence of Cas9, either no colonies or only green colonies were observed (Fig 3D and S2 Fig). More importantly, the results strongly indicate that Cas9 has efficiently stimulated gene deletion of UA08_00425 in these experiments. This conclusion is substantiated by the results of two control experiments. Firstly, no transformants were obtained when the circular gene-targeting substrate was transformed alone into *T*. *atroroseus* (Fig 3D) indicating that the specific Cas9 induced DNA DSB is required for integrating information from the circular gene-targeting substrate into the UA08_00425 locus. Secondly, no white transformants were obtained with pFC574 carrying only the *hph* gene. This control experiment shows that white conidia spores are not due to the presence of hygromycin *per se* (Fig 3E). Finally, we note that integration efficiencies are approximately 10-fold more efficient with protospacer 1 and 2 as compared to the efficiency obtained with protospacer 3.

Next, we streak purified six white transformants on solid medium without hygromycin selection. In all cases, colonies remained solid white showing that the white phenotype of transformants could be stably propagated as expected from a strain containing a permanent gene deletion (Fig 3F). In agreement with this conclusion, we confirmed that UA08_00425 was eliminated in all six purified strains by tissue-PCR (S3 Fig and S5 Fig). Since deletion of UA08_00425 results in white conidia spores, we have named this gene *albA* (S1 Appendix).

The efficient CRISPR-Cas9 mediated deletion of *albA* in *T*. *atroroseus* prompted us to identify the genetic origin of ZG-1494α and its derivative talaroconvolutin A (Fig 1).The structures of these compounds appear to be fusions of highly reduced polyketide moieties to tyrosine residues, similar to what is seen for example in cytochalasins and chaetoglobosins [16]. Therefore, we suspected that a homolog of *ccsA*, the PKS-NRPS-encoding gene linked to cytochalasin production in *A*. *clavatus* [17], is responsible for the biosynthesis of a common backbone for these compounds. In support of this view, the nitrogen-containing tetramic acid moiety present in both compounds is a common structural feature for several known PKS-NRPS products such as preaspyridone, pretenellin A, prepseurotin, as well as niduclavin, and niduporthin [9,18–20]. Due to the structural resemblance between the niduclavin backbone and the backbone of the talaroconvolutins/ZG1494α, we blasted the *ccsA* gene against the *T*. *atroroseus* genome, to identify PKS-NRPS-encoding genes in *T*. *atroroseus*. Based on this analysis we selected the gene with the highest sequence identity, UA08_04451 (ID: 46.0%, 96% query coverage) for deletion.

**Fig 4:**
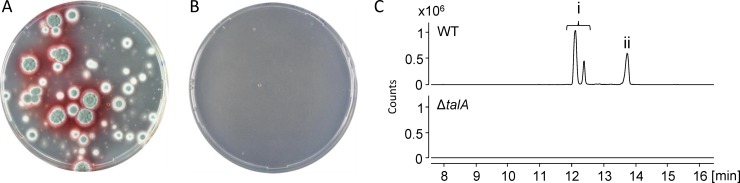
Deletion of UA08_04451 (*talA*), encoding a hybrid PKS-NRPS, in *Talaromyces atroroseus* using CRISPR-Cas9. A) Co-transformation of gene-targeting plasmid pD-hyg-talA with a CRISPR-Cas9 vector. B) *T*. *atroroseus* transformed with pD-hyg-talA in the absence of a CRISPR-Cas9 vector. C) UHPLC-HRMS analysis of the extracts of wild type (WT) *T*. *atroroseus* along with a *T*. *atroroseus talA* deletion strain. Shown are EIC @ 528.3084 (±0.0100) for ZG-1494α (i), and EIC @ 510.2979 (±0.0100) for talaroconvolutin A (ii). We suspect the two peaks in (i) to represent both ZG-1494α and talaroconvolutin B. Note that EICs are based on [M+Na]^+^ adducts.

A gene-targeting substrate for deletion of UA08_04451 was constructed and co-transformed with a CRISPR-Cas9 plasmid carrying a sgRNA targeting UA08_04451. After approximately one week, green colonies appeared on solid hygromycin selection medium (Fig 4A). Importantly, no transformants appeared when the gene-targeting substrate was transformed without a CRISPR-Cas9 plasmid strongly indicating that formation of the transformants required Cas9 activity (Fig 4B). Eight colonies were streak purified on solid CYA medium supplemented with hygromycin and were subsequently analyzed by tissue PCR. The PCR results confirmed the deletion of UA08_04451 for at least seven out of the eight streak purified candidates (S4 Fig and S5 Fig). The seven UA08_04451 deletion strains were analyzed by UHPLC-HRMS and in all cases production of both talaroconvolutin A and ZG-1494α was abolished (Fig 4C). Together these results strongly indicate that talaroconvolutin A and ZG-1494α are formed from a common PK-NRP backbone synthesized by a PKS-NRPS fusion enzyme encoded by UA08_04451, and we have therefore named this gene *talA* (S2 Appendix).

## Conclusions

In this study we have used CRISPR-Cas9 technology to genetically engineer *T*. *atroroseus* and used it to explore the secondary metabolism of this fungus. Specifically, we have identified a novel gene encoding a hybrid PKS-NRPS, which is responsible for production of medically relevant ZG-1494α. To the best of our knowledge, this represents the first example of reverse genetic engineering of a *Talaromyces* species. Importantly, the fact that our CRISPR-Cas9 system, which we have originally developed for gene editing of *Aspergillus* species, can be used without any modifications to engineer a phylogenetically distinct species, raises the possibility that it can also be used directly in a wide range of other fungal species.

## Materials and Methods

### Strains, genomic DNA, and media

*T*. *atroroseus* strain IBT 11181 was obtained from the IBT Culture Collection at Department of Biotechnology and Biomedicine at Technical University of Denmark. It is also deposited in the CBS collection at CBS-KNAW, the Netherlands, as CBS 123796 and CBS 238.95. The *T*. *atroroseus* genome sequence has been deposited at DDBJ/ENA/GenBank under the accession LFMY00000000. The version described in this paper is version LFMY01000000. DNA sequences of *T*. *atroroseus* genes *albA* and *talA* are presented in S1 Appendix and S2 Appendix, respectively. Genomic DNA (gDNA) from *T*. *atroroseus* was extracted using the FastDNA^TM^ SPIN Kit for Soil DNA extraction (MP Biomedicals, USA), and *T*. *atroroseus* gDNA was used as PCR template for amplification of the up- and downstream fragments for deletion of *talA* (UA08_04451) and the green pigment gene (UA08_00425). *T*. *atroroseus* was cultivated in liquid- and on solid CYA medium (Czapek yeast autolysate) supplemented with 300 μg/ml hygromycin B (Hygrogold, Invivogen) when needed. *Escherichia coli* strain DH5α was used for plasmid propagation.

### Vector construction

PCR fragments were amplified using the PfuX7 polymerase [21] with primers purchased from Integrated DNA Technology, Belgium (S1 Table). Construction of vectors was carried out by Uracil-Specific Excision Reagent (USER) fusion of PCR fragment into compatible plasmids [22]. The deletion plasmids pD-hyg-talA and pD-hyg-albA were constructed by amplification of approximately 2-kb up- and downstream fragments followed by cloning into two distinct *Pac*I/*Nt*.*Bbv*CI USER cassettes located on each side of the hygromycin resistance gene. The sgRNA was introduced into the CRISPR-Cas9 vector pFC330 via the tails of two primers as described by Nødvig et al. [4]. Plasmids were purified using the GenElute^TM^ Plasmid Miniprep Kit (Sigma-Aldrich), and verified by restriction analysis. A list of all plasmids from this study is presented in S2 Table. Deletions were achieved using the CRISPR-Cas9 system described by Nødvig *et al*. [4]. A circular deletion plasmid (gene-targeting substrate) was co-transformed with an AMA1-based CRISPR-Cas9 vector containing the guide RNA and the *Streptococcus pyogenes cas9* gene codon optimized for *A*. *niger*. The CRISPR-Cas9 vector also contained the *pyrG* auxotrophic marker; however, only the deletion plasmid, containing the hygromycin resistance gene, was selected for during transformation.

### Protoplastation and transformation

Protoplastation of *T*. *atroroseus* was achieved using protocols described previously for *A*. *nidulans* [23,24]. For transformation, 2.5–3 μg DNA of the deletion plasmid and 2.5–3 μg DNA of the CRISPR-Cas9 vector were mixed with 100 μl protoplasts. 100 μl of a solution of 40% PEG in 1 M sorbitol, 50 mM Tris, 10 mM CaCl_2_, pH 7.5 was added and the sample was incubated on ice for 15 min. Another 500 μl of the PEG solution was added followed by incubation at room temperature for another 15 min. The mixture was then added to 8 ml molten soft (0.8% agar) CYA medium supplemented with 1 M sorbitol and spread on solid CYA plates supplemented with 1 M sorbitol (2% agar). The plates were incubated O/N at 30°C, and the next day overlaid with 8 ml soft CYA medium supplemented with 300 μg/ml hygromycin. The plates were incubated at 30°C until transformants appeared on the transformation plates (approximately 1 week). Transformants were re-streaked on CYA plates containing the same antibiotic concentration. Tissue-PCR as described by Nødvig *et al*. [4] was used for strain validation (see S3 Fig and S4 Fig). Two sets of primers were used to validate the deletions of *talA* and the green pigment gene. In one reaction the reverse primer would bind in the promoter of the marker (P*gpdA*) while the forward primer would bind outside the upstream targeting sequence. In another reaction designed to check for negatives or possible heterokaryons the forward primer would bind in the upstream targeting sequence while the reverse primer would bind inside the gene.

### Chemical analysis of *T*. *atroroseus* strains

Validated *T*. *atroroseus* strains were grown for 7 days on CYA plates and plug extractions were performed as described by Smedsgaard [25] with the exception that secondary metabolites were extracted with 3:1 ethylacetate:isopropanol containing 1% formic acid. Ultra-high Performance Liquid Chromatography-High Resolution Mass Spectrometry (UHPLC-HRMS) was performed on an Agilent Infinity 1290 UHPLC system (Agilent Technologies, Santa Clara, CA, USA) equipped with a diode array detector. Separation was obtained on an Agilent Poroshell 120 phenyl-hexyl column (2.1 × 250 mm, 2.7 μm) with a linear gradient consisting of water (A) and acetonitrile (B) both buffered with 20 mM formic acid, starting at 10% B and increased to 100% in 15 min where it was held for 2 min, returned to 10% in 0.1 min and remaining for 3 min (0.35 mL/min, 60°C). An injection volume of 1 μL was used. MS detection was performed in positive detection on an Agilent 6545 QTOF MS equipped with Agilent Dual Jet Stream electrospray ion source with a drying gas temperature of 250°C, gas flow of 8 L/min, sheath gas temperature of 300°C and flow of 12 L/min. Capillary voltage was set to 4000 V and nozzle voltage to 500 V. Mass spectra were recorded at 10, 20 and 40 eV as centroid data for *m*/*z* 85–1700 in MS mode and *m*/*z* 30–1700 in MS/MS mode, with an acquisition rate of 10 spectra/s. Lock mass solution in 70:30 methanol:water was infused in the second sprayer using an extra LC pump at a flow of 15 μL/min using a 1:100 splitter. The solution contained 1 μM tributylamine (Sigma-Aldrich) and 10 μM Hexakis(2,2,3,3-tetrafluoropropoxy)phosphazene (Apollo Scientific Ltd., Cheshire, UK) as lock masses. The [M + H]^+^ ions (*m*/*z* 186.2216 and 922.0098 respectively) of both compounds was used. Extracted ion chromatograms were used to evaluate the production of ZG-1494α and talaroconvolutin A.

## Supporting Information

S1 Appendix DNA sequence of *albA* including 3 kb up- and downstream sequences. (DOCX)

S2 Appendix DNA sequence of *talA* including 3 kb up- and downstream sequences. (DOCX)

S1 Fig The stability of AMA1 plasmids in *Talaromyces atroroseus*. (DOCX)

S2 Fig Deletion of the green pigment UA08_00425 (*albA*) in *T*. *atroroseus*. Second independent trial.
(DOCX)

S3 Fig Tissue PCR analysis for verification of *albA* deletion. (DOCX)

S4 Fig Tissue PCR analysis for verification of *talA* deletion. (DOCX)

S5 Fig Tissue PCR analysis for verification of primer functionality. (DOCX)

S1 Table List of primers. (DOCX)

S2 Table List of plasmids. (DOCX)
