# Supplementary material for: Genes Linked to Production of Secondary Metabolites in Talaromyces atroroseus Revealed Using CRISPR-Cas9
Source: PLoS One. 2017 Jan 5;12(1):e0169712. doi: 10.1371/journal.pone.0169712 (PMC5215926; doi:10.1371/journal.pone.0169712)
Supplement: S2 Appendix — (DOCX) [file pone.0169712.s002.docx]

**S2 Appendix. DNA sequence of *talA* including 3 kb up- and downstream sequences.** The coding sequence is highlighted in yellow, while predicted introns are marked in green.

CCAGCGTTCGAAATATAGTCAATCCCACTGTGCCAACGACGCACCATCCAATGGCAGCAGCTTCGTCCCAGGCCATATCATCCGGTACTCTCCACAGTTGGTCTGCCCACACTGCGATGTATTCTGCAAACGCGCCAGACTGAGGATCTAGACGGTTCGACGCATGCACGGCTCCTGCTACCCGGTCGCCTATCTTAAGAAAAGACCTGTCTACGCTGTCGCCCAAGGCAATGACGGTGCCTGCATAGTCCGAACCAACACCTGCACCAGGACATGGGAAATTGGTGGGCATTTTCCAGTCACATGGGTTTAGAGCCACTGCTGCTGTCTTGACGAGCACTTGGCCTGGCAGAACGCTAGGAAGACCTCGATTGTGACATAGGGTAAGTGAGCCTGGTTCTTCAGCTTGTATGACTGCTCGCTGGATTGTTGGAACATCTAATTTAGCCGCCATGATTGATCGGGGGTTGGAAGGTAATAAAAGCGTATTGTGCACGGCGGTAGGGATACTTCAATCAATGAACTCCCACTGAATATCTTTTGAAGCGTGACCGACTAGTTTATCAACGTCAAACTGACTTGGCACCTTTCTGTATATCTATCCTATCTCTACGGTCCCGAATATACTTGCGAACATACACTAAAGAGGAGGCTGTATTACTTTCTTATTTGTCTTCATGTTTTTTTTTTTTTTTTTTTTTTTTTTTTCAACTCTTTTGATCCACTTCCCAACCAACGAGCATAGTATCTCCGAATATCAGCTGCAAGAGGCTTGCTCCGAATATGAAATTCGGACCAGGGAAACGCTCCGAACTGAATATTGTCAGAACTGATTTCCTATCTGGACAATAATTACAGGGCAATTCTAATTTCTGATGAGGCAGCCTATTGCAGAGGTAACCAAGTTGCTGTTTGATAACATTTTTCTTTCTTTTCACTTTCTGTTTCTTTCTTTCCGGTCATTTGTCTCACAAATTGTCCTTGCAAAGTCCTGTCGATAGTATGTATTACTTTCCGGTCCTTCTCACATTACTTTCATCTGGTCTCGCGGAGCAATGGCGGCAACAGCCCCTAGGAGATCTATTTTCAGGGCACTGGGAGAGTCCGTTGGGGGGATTTGGGCTCTCTCTATCAGAATGCAAAACCAGCCATACAACAGCATATCAGCAAGGGTATGGGCCGGTAGAATTCTCTCTCTCAACGACAGAGCATCTAGAGGTACCAAAACTATCGTCCATCAACGCCACAGCATGGGAACAATGGGAATTTGACGGTGTGTCCGAGTCTGGCAACGCCGGATTGATTGTGGGGTTCTCGCGTGATGCCTCCTACGCCTTCTTTGGTCAAGGCAATCTCCGCGTTGAGTTTTTCATGATTTTGGAGGATGGAACTGTCATTCAAGAACTTGACTACCTACAAGAATCGACCATTATTGTCTGTGATGACACCGTAACGGGCATCTGGAATAGTACCGGCCGCAGTTATTCCTTCCAGATCTCCAAGGACATGTCTCAGGCGAAAGTATGGTGGGATACTCCTCAGGCACAAGGAAGCCTGTCTCTACTGTCCCAATCGCCCCCGCATCTTGCAGATGGGTCGCTGTGGCCTGCTGCTGACAAGAATGCAACAACTGAACTCGGGCCTGGGTTACACTTCAATCAACCCATCGCTGGCGGCCGTATAGAGGCTGATGTCACGATAAGAGAGCAGCGGATCCTTGTCTCGGGAACCGGTGGACATGGCCGCCTCTGGGCCAAAGATGGTTGGTTCCAGATATGCAGTGGATGGTTCATTGTTCGTGCCTCTGCAGGGCCATATGTCCTTTCCTACTGGGAGATCGTATCCCGAATCAATCCAGGTGTCTCATACTATTCTGCACAATTGTTCAAAGAAGGTGAATTGCTAGTCGCAACGCAGATTGGAGAGCGATCTGATGCTCAGGACCACGTTCTCTATACACAGGAGCTCGGAGGCAATGTGACCGGACTCCTCAGAGACCGCTCCACCGGCCATGTGGTCGAGTTTGTATCTCCAGTGCAAAGAAAGAGGTGGCGCTTCCTTGTTCGAAATAAAAGAAAGAAGTTCGAGATGGGCATGGGTGGCGGCTCTGGCCTCACAGGATTCACAAATATTGTGACGGGAGGAGAAGTCTGGGAGAAACCATACGAAGGACGCGGATTCTCAGAGCAAGTGACCTTGCCGGACAAAATCAAACAATGGCAAATTTGGCTTGTCTACGGTATTGGCTTCCTCAACAAGGGGAAAACGTCTTTGATCAAGATGATTGGATATATAATTTGATGCGGGTTTGGTTCAATTTTGGATCGTAGAACGCGAATGGTAACTGTAGTTAAGTCGAATGATATGCTGGTTACGTAAATGAATCATGCTACTCGGTAGAATGATTCTGTGGGTTGATGAAGCTTTATATCTCTAAGCTATTCTCATATGGTGTTGTCACAATGGTTTCAGGATGACCACGCTTTTTGACACTTAAAATCAACCAGCTCTGATACTTCTGTACTTTATGATACCCAAGGTTATCGCCATCTACCCCAAATGACTGGAATCCTGTTACCCCTGGCAAAGGAAACAGACAAAAGAGAAATTTTAGCCTATATTCAATCATCCATCTCAGCTCTATGGTACGACATCGGAGATTCTCCATCATTCGGAGCCCCTTCATGATTCGGAGAGTTTGAAGAATGGCAGAATATCGGTCCGAAACTGCGGGATGATTAGTATTTGTATGTAATAGAAATATCGGAGAAGGTTGGTGTTGACCCTCTGGTGGTAGTTGGATCTTAGAATAGTACATTATGGTCAGATTAGATGCTATAAACCTGGACCTTCTTAATTTCAACTCAAGTGATCTTTATAGTGGCCTTACTCCTTAAATTGATCTACGGTTATACCTAGGTGTATTTGACTTTTCTGATTTTATATATTCTGTCATTAAATAATCTATAAAAAAAAAAAGGCCTTCCAAGAGCATCCAACATGGGCACCATCCATACGCCTATTAAAGAGGCCAATGAGCCAATTGCCGTTGTTGGTAGTGCCTGTCGATTCCCGGGATCATTGAATACTCCATCCATGCTTTGGGATTTTCTGTGCAAACCTCATGATCTCCTTACCAAAATTTCCAACGAAAGATTCAACCCAGACGCATTCTACCATCCAGATGGAATGCATCACGGTACCTCTAACGTGAAGGAGTCATATCTCCTTCGTGAAGATCACCGCGCCTTTGACGCCGGCTTCTTCAACATTAAGCCTGTCGAAGCTCATTCAATTGACCCACAACAAAGGTTGCTGATGGAGACAGTCTATGAGTCTCTCGAGGCTGCCGGAATGTCTATAGAATCACTAGCTGGCTCACAGACGGGAGTATACGTAGGCTTGATGTGCGGTGACTTCAGTGAACATCTCCAACGTGACCCTCAGTCATTGCCTACATATATGCCCACAGGAACGGCTCGCAGTATCATATCCAACAGGGTGTCTTACTTCTTCGACTGGCATGGACCATCAATGACTATTGATACCGCCTGTTCTTCTAGCTTAGTTGCTGTCCACCAGGCAGTGCAGCAGCTGCGAAGCGGCGAGTCAGATGTTGTTATTGCAGCTGGTGCCAACCTTATTCTTGGACCTGAGCTGTACATTGGGGAGGCCAAATTGAAAATGTTATCTGCCGATAGCCGGTCGCGAATGTGGGACGTCGATGCTGATGGATATGCACGAGGGGAAGGAATTGCCTCGGTAGTTCTGAAAAGGCTCAGTGCTGCTCTTCAGGATGGTGATCACATCGAGTGTGTGATCCGCGAAATTGGAGTCAATCAAGATGGCAGAACAAAAGGGATCACAATGCCAAATGAATTGGCTCAAGCTGACCTGATAGAAAGAACTTACTTGAAGGCAGGCTTGAATCCTAAAGACAAGAACCAGCGCTGTCAGTTTTTTGAAGCCCACGGGACTGGCACTTCAGCAGGCGATGCTCGTGTAAGTCTTGAGTTCCCTCTCTCCCCCCGCCCATTTTACAGTTCCATATATCAAAGCATGATAGCTGAGACAATAATAGGAGGCAGAGGGTATTAGTAGGGCATTTTTTGGCCATGAATTTGGCAATTCTGACGTAGAAAATGTGCTGTATGTGGGCTCGATCAAGACAGTGATAGGTCATACCGAAGGCACTGCAGGCTTAGCAGGGCTCTTGAAAGCCTCCCTCGCCGTTCAGCATGGAATCATCCCACCAAATATGCTCTTTAATTCTCTCAACCCAACAGTCAGCCAGTTTTATAATAACCTAGAAATTGTGACAACCGCTAAACAATGGCCCAAAGTCGAAGGACCTCGCCGAGCCAGTGTCAATAGTTTCGGCTTTGGGGGCACAAATTGTCACGTGATCGTTGAGAGTTTTGAAGATTCAAGCAGCGAACACGAGCAGGTGAAAGAAGTGCAAGTGCCTTTCACTCCTTTTGTGTTTTCTGCTGCATCTGAAAAAGCGCTAGAGGCAAATTTAATGGCCTATTCATCTCACCTTCGATTGAATCCCGATCTCAATCTCGGTGACTTTGCTTTCACGCTACACTCAAGAAGATCAGCGCTCGGGGTACGAGCAGCCTTTGCAGCTAGGTCTTCTGATAGCCTCTGTGCGGCTATAGATGAACGCATTCAGCTCCATAAGACGGACACGAGCACGCCACTCGGTGTCAGACCTAACACCGCTGCTCCATCTATCTTGGTTGTCTTCACTGGACAAGGAGCGCAATGGCCAGAAATGGGACGTCAACTGATATTATCATCGGCCTTTGTTCGTAAATCCCTAGAAGAACTGGATCAAACGCTTCAAGATTTGCCAGATGCAGATAGACCTTCATGGTCTCTGGTGGAGCAGCTACTTGCTGATCGCTCTCACTCGCGCGTTGGAGAGGCTGCCATCTCACAACCCTTATGCACTGCTCTTCAAATTGTTCTAGTAGAACTCTTAAGAGCTGCTGGGATTAGTTTCAAGGCTGTTGTAGGTCATTCATCAGGTGAGATTGCCGCGGCATATGCAGCTGCGTTCATTTCTCGCGAGGATGCGATCAAGATTGCATTCTATCGAGGTCTAGTCGCGAAATACGCGGGACAAGATCGACCAGGCGCGATGATGGCAGTGGGGACATCATTTGACGACGCCAATGACCTCTGCGCTTTGGACACCTTTGAAGGTCGTCTTTCTGTTGCGGCGTGCAATTCCTCATCCAGTGTGACTTTGTCTGGAGATGCGGATGCCATTGAGGAGGCTAGGTTCATTCTTGCTGAAGAAAAGAAATTTGTCAGAACCATAAAAGTGGACAAGGCCTACCACTCTCATCATATGCTTCCTTGTGCGGAGCCCTACGTTAGTGCGCTTCTTGAGTGTAATATTGAGCCGAAACAGCCAAAGACTGGCAGTTGTCACTGGTACTCGAGTACTTACCAGGGAATTAAGATGGAGGCTCAAGACGAGCTCAAAGGAGTGTACTGGAAAGATAACATGGTGAACCCCGTGCTCTTTTCACAGGCTATAGAGGCCGCAGCATCCAATGAGGATCCTTTCAATCTTGTTATTGAGGTTGGTCCACATCACGCCTTAAAAGGACCTGTTCTTCAAACATTGCAAGATACTTATGGCCAATCTATCCTTTATACGGGCCTCCTCAGTCGGGATAAACATGATGTGGAGAGCTATGCCAATGCACTAGGTTATTTTTGGAGCCACTTCAGTCCGACTGGTGTCGAACTTGGCCAGTACGACGATTGTCTCTCTGGAGGTCAAAAGAGACAAATTATACCCTCATTGCCGTCCTATCAATGGGATCATGACCGGACCTTCTGGCACGAAACCCGCTTATCAAGAGCTCATCGTAACCGCAAGGATCCATACCATCCTTTACTGGGTTCGAGAACACTAGAAGGGACAGAATCAGAAATGCGCTGGAGAAATATACTTCGGCCTAGCGAACTTCCTTGGGTTCATGGACACCAACTACAAGGCCAGATGGTGTTTCCAGCGACAGGATATATTGCCACAGCTATAGAAGCTGCAAAATACATGGTCAAAAACATCCCTATATGTCTAATTGAAGTGTGTGACTTTGTTATTGGAAAGCCCTTAACGTTTGATGACGACCAGTCCGGCGTTGAGATGTTGTTCACATTGTCCGATATTTCTAAGAACAGTTCGGGGTTGTATTCAGCCTCATTCAGGTACCACGCATGGACAAATCAGGAGTCAGATACATTGTCATTGTTAGCCTCTGGCTGCATTGTGGTAACTATAGATGAGACATCATCTTCGAATGATGTGCTTCCACCACGAAGTCCTGAACAACCGAATATGGCATCTGTGAGGGAGGATCAATTCTACGCTTCTCTTGACGACCTTGGTTATGGCTATTCTGGGGATTTCAGATGTCTTTCATCAATGAAGAGGAAGCTTAACTACGGTTCCGCCTACGTATCTGTGCCACAGCAGGAAGCCGTTGACGCTGTTCTTGTACATCCCGCCTTCCTAGATGTCGGCTTCCAGTCCATATTCCTGGCCTACGGGTGGCCCAACGATGGAAGCCTTGATCAACTACATGTCCCTAGAAGCATCTCAAGGATCCGGATCAACTTTGCTCTCTGCCAGAAGGACTTAGTTCCTGGGACACAGCTACAATTGGACTCTCACCTGACAGATAATCCGTTGACCGCGGGCGCTATACGTGGGGACGTTGATATATTTGGAGCTGATGGACAATCCACCCTTATTCAGGTTCAGGGTATACAAGTGGTTGGACTTTCAGATGGAAGCCCTCAGTTAGACCGGCAATTGTACTCAGAACACATTTGGGGCCTGGCAATCCCTGACGCTCAATGCGCAGCAGATAATCGCGCAACACCGGAGGATTACGAGCTTGCATGGAGTCTGGAACGTGTATCAATCTTCTACATGAAGAAATTACAGGCTGATATCAAGCCAGAGGAAAGAATAAACCTGGAATGGTATCACCATGCACTTTTCGATTTCGTTTCTCATATTCTTTCCAAGACAGAGAAAGGCAAACAACGTTTTGCAAAGAGAGAATGGCTTGACGATACTTGGGAACAAATATTAGAGATCATGACACGGTAAGTACCACTCCATATTTCTCCATGACCTTATAGGTACCTATGCATGTGCCAATTTCTCATACCTTGCACTAGATACCCTGATAGCATAGAAATGAAACTTACTCGTACGGTTGGCGAGAATCTCGCAGCTGCTGTTCGAGGCGAGACAAACATTTTACAGCATTTACTCGCCGACGGTCTCTTAAACAAGTATTATACGGACGCGATGGGTCTCAAAGAAACAACAGAGTTCCTAGCCAAGTCTGTTGCCCAGGTTGTGCATCGCTATCCACATATGAAGATACTTGAGATTGGTAAGAAAATATATATATATCAAATAATTCTATTTGTTAATACCTTTGTGTTCTAACTTTGCATCTTAGGCGCCGGTACTGGTGGAGCCACGAAATCCATCATGAGAGACATCGGCCGGTCCTTCTCTTCTTATACATACACGGACATTTCAACTGGTTTCTTTGAAACTGCCAAAGGAATCTTCGCTCAGAATCTGGACAGAATGATTTTCAAAGCTTTGGATATAGAGAAGGATATACTTGAACAGGGCTATGCAGAGCATTCTTATGACCTTATCATAGGATCCTTGGTTCTACATGCTACTTCTAGCCTGCGGAAAACGATGGAAAATACACGTCGACTGCTGAAGCCAGGAGGCTATCTCATAATACTTGAGATAATCAGCAATGATGTGATCAGAACAGGATTTGCTATGAGCGGCCTGCCTGGTTGGTGGTTGGGTAGAAATGATGGCCGTCGTTTCTCTCCGTGTGTATCTTCCGCTGAGTGGCATAACCTGCTTGTAGAGACCGGGTTTTCAGGAATAGACTCACTCAGCCCTGAAACTGACATCTTACCACGTCCTTTGTCTGTGATCATATCTCAAGCTGTGGATGATCAGGTCAATCTTCTCAGGGAACCTCTTCAATATCCAGGCCAGACACTAGGGAACACACAAGATTGGGATCTTGTAATCATTGGTGGAGAGACGCTCCGAACGATAAAACTAATAGATGAAGTAATCCGCCTGCTTCAGCCCTGGTACTTGCCAGTTACACGAATCCGCTCTCTGCACGATACCGAACGCTTGTCCAATATATCAGCCAAGAGTGTCGTGCTAAGTGTTACAGAGCTCGACCAACCCATATTCCTGGATTTCAGCCCTGAGACTATGGAAGGTCTCAAAAGGCTACTTGGCTATCAGCGAACCATTCTCTGGGTCACCCAAGGTTGCAGAGTTGATGAACCTTACATGAATATGACCGTTGGGTTTTGCCGAACCCTGGCCCTTGAAGCTCCAGATACCCAGCTGCAGCTCCTAGATCTAGACATATCACGTAGGCCTGATGCTCGACTGCTTGCCGAGTCACTCTTGAGATTAAACTTCACACAGGTTCCCGAGGTCTTGTGGTCCGTTGAGCAGGAGATAGTCCAAGAGGCTGGGAAACTGTTAATTCCGCGTCTTATTTTGAATCAAGAGCAAAATAATCGCTACAATGCCGCAAGAAGGACCATATTCGACACCAAGGATGTGCGCCAAACTCCTGTTATTCTACAACCCACACCCGTCGGTTATAGTCTTGTCAAGCACGAGCTGGATTTGTGCCCGAATGACGTTCAGGTCGCAGTTAGTCACTCAACGTTGGTACCGATTGCAAATTCAATGTACGGTATTGCTGGGATAAATATATCCACTGGCAAGAGCGTTTTGGGCTTCTCCACTATCAATGGTTCCCAGCTTGCTATAAATACTGACAGGCTTATTGAATACAATTCATCCAAAATAGGGGAGAGCAGTCGCCTGATCTCTCTTCTGTTGGTTGAGATGGAGGTGGACAACATCCTTTCAGTTTGTACATCCGATGAGCATATTCTGCTCCATCAAGCATCTGCAGAATTAGCGGAAAGACTTGGAGAGCGGGCCCATGAGAAAGGGCTCAGAATATCCTTTACTACGTCTCATGGAAAGCCTCTGGACAAGACCTGGATTACCGTCCATCCATGCTCTCCACTACGTACTATTAAGGCAACTGTGCCTCTAGAAGTTTCGATTTTCATTGATTATTCAACCAACGACGATGGCGTTGGTGAGTTAATATCGTCCTGCCTTCCTGATACGTGTTGGCGAATTACATCTACGGACGACAGATTTCGTACCAGAATATCGGGGCAAAAACTTTTTGAGAAGCTCAAAGATTTCATTAAACGCGTAGGGACGGTAGACATTGATTCTTCCAGCCACTCGTCCCCGGTTCAAATGAGTCCAATGGATATTGCAGGGAACGGTAAAACTGCAAGCTCCACGATTGTCAATTGGAGTGGCACCACTGAGGTCTCAGTGAAGTTGTCATCTATCGATAGCCAAATCAACTTTGCACCTGACAAGACATATGTGTTATTTGGGTTGACAAGCGATCTGGGACAGTCACTGTGTGACTGGATGGCTGCTCATGGAGCCCGGAATATTGTCCTAACTAGTAGAAACCCGAAGATCGATCAAAGGTGGTTGGACCAAATGAAACGCACGGGTGTGAGGTTGCAAGTCTTCTGCAAGTAAGTGAGCCTCTCTTTCCTGGGGAAAAAAAAATGTACGCTGTCAAGAAGCTAACTTACGTTTTACAGTGATATAACCGATAAAGTGGCAGTCCAAGCAATTGTTTCAGAAATACGTGCCACATTCCCACCAATTGGGGGTATTGCCCAAGGAGCAATGGTATTGGAAGACGCTTCATTCTTTGACATGTCATTTGAAACTATGGACAAGGTCTTGAAACCGAAAGTACTAGGTAGTATTCACCTTGATGAGCTATTCCAAAACGATGACCTTGACTTCTTCATCTTCTTCTCATCACTAGCATCGGTAAGCGGAAATCGCGGTCAATCCAACTACAGTGCTGCGAACATGTTCATGACCGCCTTGGCTTTTCAACGTCGACGAAAGGGGCTCGCCGCGTCTGTTCTCCATATCGGTGCCATTATGGGTGTTGGCTACGTTATGAGAGAAGTGAGCGAAACCGTCTTTCCTGCGATCCGTCGTGCCGGGTTCACGTGGATGCCTGAGCGTGGCTTCCACCAATGTTTCGCTGAAGCTATTGTTTCTGGACGGCCACAATCTGGTCGCAGTCCGGAGATTGTCACTGGACTCCGACTAATCAATGCTAACGAGGAAGAACCTGCTCCTTGGATGAATATCCCCAGATTCCAACATTGCATTGACCGCAGTGGTACGGCGGGACTTAGGGAGGGTCATGGAAATGCAGTCGTAGCAGTTAAGACGCGGCTGCTAGTGGCATCAACTCGGGAAGAAGCCCTGGAGATGATTCAATGTGAGGCAGTGTCAGTACATCTCGTCAACAAACAGCTAACAAGCGTCAGATGCATTCTTCGCAAAGCTGCAGGCAGCTTTGCAGCTTCACATCGACGATCCTGCCGTTCAAAGGCAGGTTCTGAGTTCTGGGGCGGATGAATTGGGCTTTGACTCTCTGGTTGCTGTTGAAATTCGGTCATGGTTCCTCAAAGAACTTGAAGTCGACATGCCTGTGCTTAAGATCCTTGGCGGGGCCTCTGTCTCAGACCTTCTTATTTTCGCCCTTGATAAGCTGCCTGATGAACTAATCCCAAAAGTTGCGGCGATCCCTGAAGCTGTAGTAAACACGAGCAAAATCAAGAAAGATACGGTGACACCGCATTTCTCGCCATCAAGCGTTATTAGCTCAAAAGCAACCAGTGATGGGGAAAGCCTCTTCTCGCCAGTCAATGGCTCTTCCCCTCCACAGAACTCTGTTGTCTCCCTTGTGTCTTCGTCGTCCTCTACGTATGACAAGGAAGAGCATTTCACCCCGAATTATGAGCCTAAGTTTGAAAAGGTAGTGCCATTATCTTTTGGACAGTCACGATTTTGGTTCTTAAAGCATTATCATGAAGACCAGACAACGTTCAACATTACATTCTCGACTCGACTTAAAGGTCCAGTGCGAATTCGTGACCTGGAAAACGCGGTGAGAACGATGGGCTATCGTCATGAGGCACTGCGAACGTGCTTTTTTGTACAGGATTCCAACCAGCAGCCGATGCAGGGAATTCTAGAGAAATCTTCCCTGTGTCTGGAGAAAATGAACATTCAGAATTCAAGTCAAGTCGCGGAAGAGTTTGAAGCAATGAAGAATCATGTTTTCGACATAGAGAATGGAGAAATTATGAGGGTTATTCTCCTGTCCCTTAACCCGTTAAATAGCTTCCTCATTATCGGTTACCACCACATCAATATGGACGGTGCAAGCCTGGAAGTATTCCTGAGGGACCTTGAGTTGGTCTACTGTCGTAAACCACTGAGTCAACCAGTATACCAGTATTCCGCTTTCTCGCTATGGCAAAGAAAGGAAATCGAATCCGGCAAAATGAAAGACGAGCTTCAGTACTGGAAGTCCGAATTTGCAGATCCTCCTACTCCATTGCCATTGCTCCCTTTCTCCTCTACGAACAGACGCGAGGCAGTTTCAACATACAGTCATTATCGTGCCGATGTTCGAATTAATTCTCAGTTGGCAGAACAGATAAAGAATATGTGTTCGAAAAGGAAAACCAGTATTTACCATTTTTATCTCTCCGTATATGAGGCAATGCTCTTCCGGCTCTTGGACACGAACGATCTCTGCATCGGCATGGCAGATGCCAACCGCAGCGAAGATACCTTTGTCAATAGTATGGGGATGTACTTGAACCTGCTTCCACTACGATTTCATCTTCGCTCGAATATGCAATTCATAGATGTGCTGAAAGAAACGCGCCGTAAAGCCTACAGTGCGATGGCTCATTCTCGTCTACCTTTTGACGTTCTTCTTGACGAGCTGAAAGTACCACGCTCCACGTTGTATAGCCCATTGTTTCAAGCTTTTATCAACTACCGCCTGGGAGTCCAAGAAAAGAGAGCCTTCGGAGATCTCGAGTGTGAGGGTGAGGAATACTCGTTAGGCAGAACTGCCTACGATATCAGTCTGGATATTCTTGATAACCAAAACAGCAATACCCTGCTTATGTTCATCGTACAAACGCAGTTATATTCGGCAGAGGATGCAAGGCTTATGTGTAACATCTACGTAAGCCTTCTTGAACAGTTTTCCAAGGATCCAACCCTTCGTCTAGATGAGCCATCTCTCTTTGCAAAGGAGGATATTGCAGAGAATATTCAACTTGGAATGGGTATAGTTTCCTATCTTCTAACGATAGAATGTGGCATTCACTAAAACAATCCTAATACCTTCTAGGACCGAATCACAACTCAGAATGGCCAGAGACCATGGTACACCGCGTCTACCAAATCATAGAGACATTCCCTGACAGTATTGCTCTTAAAGATACGGAGCACAACGAGTTGACATATCGACAGCTGGCTAATCGTATAGGCTGCATAGTATCATCTCTTCTTGAAGCAAATCTAGAAGAACATGCTCCTGTCGGTGTATTTCAAGAACCTTCACCTGATTGGGTCTGTTCAATTCTTGCTGTGATGGCTATTGGAGCCATCTACGTGCCATTTGACGCGAAATCGCCTACATCTCGTCTAGTGTCGATGATGGAAGATTGCCAGCCCACTGCTGTTCTCATCCATGATGCCACGGCCTCGAAGTTTGGAGGCTTGAGGTTGTCCTCTCGGGCAACTGTGATTAATCTTGCTAACATTCCATTAGACATCTCGCACGAGAGTCGTAGTGTCGCAGCCAAAGCATGTGATCCTGCGGTGATACTGTACACCAGCGGTACGACAGGGGTACCGAAAGGGGTCATCTTGTCCCATGAGAGCCTAAGGAATGAGAATGAATTCGAGGTAGTCTCAGGTCCCGAAATTGTCCTTCAACAGAGTGCAATCAGTTTTGATCTCTCACTGAATCAAGTTTTCACGGCTCTGGCACATGGTGGAACACTTGTTATCATTCCAAGATCTATGCGGGGCGATGCAGTCGCAATAGCAAATATAATAGCCAAGGAAAAAGTCACCTATACTGGAGCAACGCCGTCAGAATATCTCAGTTGGCTTCAGTATGGGCGATCTGAGCTTTTGCAAAACAAGGCTTGGAAGTTCGCCATGTCATGCGGAGAGCAGTACCCACAACAACTCGCTAATGAATTCAAACGCCTTGAACTTCCCCATCTAAGCCTGTGGAATGCATATGGACCAACCGAAGCCACTCTTTCGTCGAACAGAATCCAGTTATTCCTGAAAGATGATGATTCAAACCCACAGCATATTCCCGTTGGTTATACACTGACCAATTGTTCGGTATATATCGTAGACCGAGACCTCAATCCGTTGCCTGCTGGAGTACCTGGGGAGGTCTGTATTGGGGGCGCCGGGGTCGCAATAGGCTACTTGAACAACAAGGAGCTTACCACAGAAAAGTTCGTGCCAAACCGGTTTGCAAATCCACATTTCCTGTCGCGTGGGTGGCAACGAATGTATCGAACTGGCGACAGAGGAGTTCTCCAAGATGATGGAGCTCTGAAGATTCTGGGTCGCATTGACGGTGATACACAAATCAAGCTGCGTGGTATTCGGATTGAAATGCAAGACATTGAGAATACCATTCTTCGTGAGGCAAACGGAGCGATATCGGATGTTGTCGTGGTGCCTCGTGGAGATCCTCCAGTCCTTATGGCTCATGCTGTCCTGTCATCCGTCTCTCCGAAGGACCAGACCGGCTTTCTCCAGCATCTAAATACATCTCTTTCTTTACCACAATATATGCGGCCAGCAGCCATTATCCCTATCGATAAAATGCCTCTGACTACTCATGGCAAGATTGACAGAAGTGCGGTGCAGATCCTTTCGATAGCCCATCCCTCCCAAAAGCCATCTCCTGTCACTGAAATCACGCCAATGGAAAATCAACTTTGGCAGATCTGGGAGAATGTCATTTCAAAGGAAGTTTTACAGTTGCACGCCATTGATGGTGATACAGACTTTTTCCATGTCGGAGGCAACTCGATGCTTCTCATCAAACTGCAAGAATTGATCAAACAGAAGTTTGGCACTTCGTTGGGTATCATGCGTCTTTTTGAATGCAGCACGCTGCGTACGATGGCAGCAGCGATTCAAGACGCGTCCGTCACCAGGTCTCCTGATATTGTTCACTGGGAAGAAGAAATAGCTTTTCCCGAAGAGCTGTCAGTCATGGCCTCGTCCCAGTCTACTAACCTTACAAGAAAGCATGCTACTAGTCCTCTAGTAATCATCCTCACTGGTTCTACAGGCTTCCTGGGGAAAGAAATACTCCAACAGTTAATTGCATTGCCAAGTGTCCAGGAAATTCACTGTGTTGCAGTCAGGGATGAGAAAAAGCTTGAGGACATGGTGACCTCCTCAGGCAAGGTAGTAATCCATCAAGGAGACTTGAGCCTTCCACAATGCGGGCTCTCCGATCAATCTGCCTCTGAAATTTTCAGCAAAGCAGACGCAATCATCCACAATGGCGCCGACGTGTCTTTCTTGAAGAGTTATCGATCCTTGAGCCCATCCAATGTCGAATCGACGAAAGAGCTGGTTCGTCTGTCACTACGCCAACGGCAACTTATCCCATTTCACTTTATCTCAACCGTCACTACAGGACGACTGAATAAGAGCGAAACTTTTGGAGAAGTCTCGTTATCATCGACTCCTCCTCCTCCAGGGTTCCCAGATGGTTACGTTGCCAGTAAATGGGCCAGCGAGGCCTTTTTGGAACGGGCAAGTACGAGTTTTAGTCTTCCGGTATACATTCACCGCCCATCGAGCATTACAGGCGAAGGAGCAAGCGATCTCGATGTAATGCACAACATGCTAAAGTATGCACGCCTTACAAAATCCGTGCCTGAATCATCCCGCTGGAAAGGATATATCGACTTTATCAGTGTAGAGAATGTCGCTCGCGGGATCATCCAGGAAGTCCTCCACAGGGAAAACGAAAGTAGTACTCCCGCAAGCAATACCGCTCATGTGAGATACATTCATCATTCCGGAGATATCGTTACCACAGTCGAGTCGTTTAAAGACTACTTGACAAATGAAAGTGGAGAAACCTTTATAGGATTGCCGCTAAGAGAGTGGGTGGAAAGGGCAAAGGAACACGGCCTGAATGTCCTAGTCGCGGGTTACTTGACAGCAAGTGATGAGAGACAGGTGGATGTGGTATTCCAGAGGTTGATTAAATTGAAAGTGGCATAAGGAATTCGTAGATCGGGGCAGAGCGGTTTCTTGAATTTGCTTATTCTCAATGGAGATTGAACATTGATATGACGAGCTTTACTATTTCCATCATCTTGAAAAAATGCAAGGTCAGAAAGAATAAGTTTATTTTTCTGTGCCAAAGGATATCCGGGCCTGCTACTTACGGCCAATTCTACAGTAGAGACCGGCCAATTCTGGCGCCCATGAAATGTAAATATGTGATATTGGTTGCAGATCTTGCCATATCTGTTATCAAAATATCAGGGCGGACTTTATATTTTTATTTTAAATATAGCTATCACCCAAATTCCATGTAATTATGTAATATACTGGACATCTCTGGCTTAAAAGAGCTAGTATTAATACCTAAAACTCTAAGCTCTGGTTCTTGAGTCCTGCCTCTCTCTAGGTTTTGTAATTCCAAAATCACCTTTCGTGCCCAGTCAAGGTCTATTATTTTGTACTGGGTTGGATCACTGGGGATAACCTCACGGAGTCTGTTCCAGACAGAAAGAGGCCAGGCACGCTTTTGCATATTCGAGAGACCATGGAACCAAGATTTCCAAGAGCTTTCTCTCCGGGAAAATAGAAATTAAGAGCGGCCATAGCGATTCTGGTAGGATGATATTATCGTTTTTCTCCAACAGAAGTCTCAATTTGACGGCGGGCTCGGGTAAAGATAGTGGCCTCGTTATTGGCCGGGAGATAGCCACAGAGGATCTGGATCCGGAAGACTCGCTGATGAATGAGCTTGCGAGAAGTGTCATGGGCGAGATAGAGGGGTTTGGGTGCTAATTCAAATCTCGTAGATTGGGTACTTGTTCCAGGATATCAAGTGGGATACGGGAGTGCACTTTGTACCAATCACACCGACTGTGGAAGGAGATGATATAACAACTTTAGGTGACGGGCCTAGGCTGCAAAGCATTTGCTCCGTACCTAATTAAAAAAAATGTAGCAAGTTTGATATTATACTATGTATTGTAAGATTGTGCGCGCTAAGAAAAGTGAGGAGGTTTGTATACTATCGACTAAAGCTCGCTATCCAACAAGAGCACATGAGTGGATTTATGCGCTGCAATCGCCATATTGGGATAGCCGTCAGGTAATCTGGTCTCTAAGCTAAGTTATTCCAAAACTAATATCATTTTGCCAAGTCAATCCTTCTTGAATCGACTTGGGTCCATGCATATAATGATTGGTTCCCATGTCTGACCCCACAGGCTGATGCAAATGTAACGACGCGTTAACCAAGCGACAAAACATCGGAACAACGTACTAGGTAGCTGGGAATGTACCTGCCACAGAATTTTGCGCAATCTCAGGTAAGGTCGGAATGCACTGGGATGCCCTCAGTTAGTTAATGCCCCGGGTATATTAGGGCCGAAATGCAGACAACGGTGGACGATGAACAAACTGTAGGCGCTCACAAGTTTCTTGTCTTTGTGGAACGAAATCGACACTCACTTCACTTGCTTGATCGCGACCGGGCCTATTTGATCGCTCAATTGGATCAAATGTCAGCCAACCAATTATCTTGGCCAAAGAGTGCCGCAGACGTGTCAGACATGTTCGTTTTCATGCTGCAAGCACAGTGGATATCCGCCCAATACCATGGCCGGCACCCAGACTCAGATTTTGCAGATAAAGAATTGTGGAGAATACGGGAGAATCGAGGAGTCAATTCTCAGGGAAAGCTTCGAGAGTATGCAAAGGAGCGTAACATGGATTATAATAGGCTTCGGGATACGGTTCAAGTTAACCAGAAAGCGGGAGCCATAGAGGCGGAGTTGGGAGTTCCCGGCTTGGGAAAGACCTTATACTCTGCCTTCAAACGACTAAGAGATCTGAAGCAAAATGAAATTGAGTCATTCTGCAGGCTATTTCGTGCTAGTGATTCGTTTGCAGATTACAAGAGCTATGCAGCTATAGCGCTGGCATTATTGGACGATTATTTGACTCTGATGCAGCGGCATGATTTAGATCAATAGATAGTGGGATAAAATGACAAACTATGCATGCTTATCGAGAAGTTCTCACCCCTTTTCCAGGTTTCTGATCGTAGGTGTTGCATGGAATGGCGCTACTTTGACGGGGACATCATCCCTCTGAATCATAATCCGTTGCCGTTCAAGAGAAAAGGTCTGGAGAACAAATAGACCATCACTTTCACTCGGCCAATTGAAGCACATATCTGATAGTATATAGGCTACCACTTTCTGAGATAACTCTAGCGGTACCAGGCATAGAGGACCTTCTAATCTCTTCCGATCGAGAGTTGTCCGGTCGAGCGTATGCGACTTATCGTATAGCTGAAACCGAACGTTTCCCCGCACAAGCCGCAGCAAGCAGATTGCTTTGTTTCGATGGTATCCGTAGGGTCCTAGCCTATAGATTGAGGGCGGTTCTGTGGCTTGGGCGCAGTTCACTAGAATGGGTTCTCCACGAACGACATCCTTTATGTCATTTCCACCAAGAAGGGAAAGAATGTAGTGCATCTATCAGCTCGGCGCGTAAGCTTGTGGAGTATATGTTTTTCACGAATAACGTACCCTTGCTTCCAATAGGTCATGCTCTACGAAGTTAACGAGAGCTTTCGATAATTGAGTCCCGTATCTGCCCCGGACACAAAGTGTGGTTGTCGAGTTCTGTTCGAGAACATCATCCAGCCACTTCAGTGCCTGGCCCGACGGAGGGTCCCAAGGCAAAACAGCATAACCTTCGTAGTTGAAAGTCATCTCCTCATCTTGTGCGTCATCCTCGGGAGCGCTCACAACGGCCCCGCGATATTTCCTCGTTGCATTCTACAGTAGTGTATGATGATTAGGTAATCATCCTTTGGCCGTGGGTTCTGAAATCAAGGCGTAGATAGGCCGCATGCGCAAGTGATGAGAGATGTGATAGTCACAGAACGTGTTTATTCTCGAATCAAAGTTTGGGACTGAGTGGAAATTGACTTAATCTCTCTCATAGGTGCTCAAGGCTAGAATATATGAA
