# Supplementary material for: Genes Linked to Production of Secondary Metabolites in Talaromyces atroroseus Revealed Using CRISPR-Cas9
Source: PLoS One. 2017 Jan 5;12(1):e0169712. doi: 10.1371/journal.pone.0169712 (PMC5215926; doi:10.1371/journal.pone.0169712)

**S1 Fig. The stability of AMA1 plasmids in *Talaromyces atroroseus***. To follow progressional loss of the AMA1 plasmid as the fungus grows in the presence and absence of hygromycin selection, we used the following experimental setup presented in (A). Step 1, *T. atroroseus* was transformed with a CRISPR plasmid (pFC332) containing the hygromycin resistance gene, but no sgRNA gene insert, and plated on solid CYA medium containing hygromycin. Step 2, Spores from the resulting transformation plate (CYA+hyg) were transferred via a single-point inoculation to a CYA plate as well as a CYA+hyg plate, and allowed to grow for four days at 30°C, forming colonies with diameters of approximately two cm. Step 3, from these colonies, spores were collected from an area of approximately a mm^2^ from positions with increasing distance from the centers of the two colonies and transferred to solid CYA medium with and without hygromycin. Specifically spores were recovered from the following distances from the center of the colony: 0, 0.5 and 1.0 cm. (B) Solid CYA medium with and without hygromycin from step 3. Reduced growth on the CYA+hyg plate as compared to growth on the CYA plate indicates loss of the AMA1 plasmid.

A


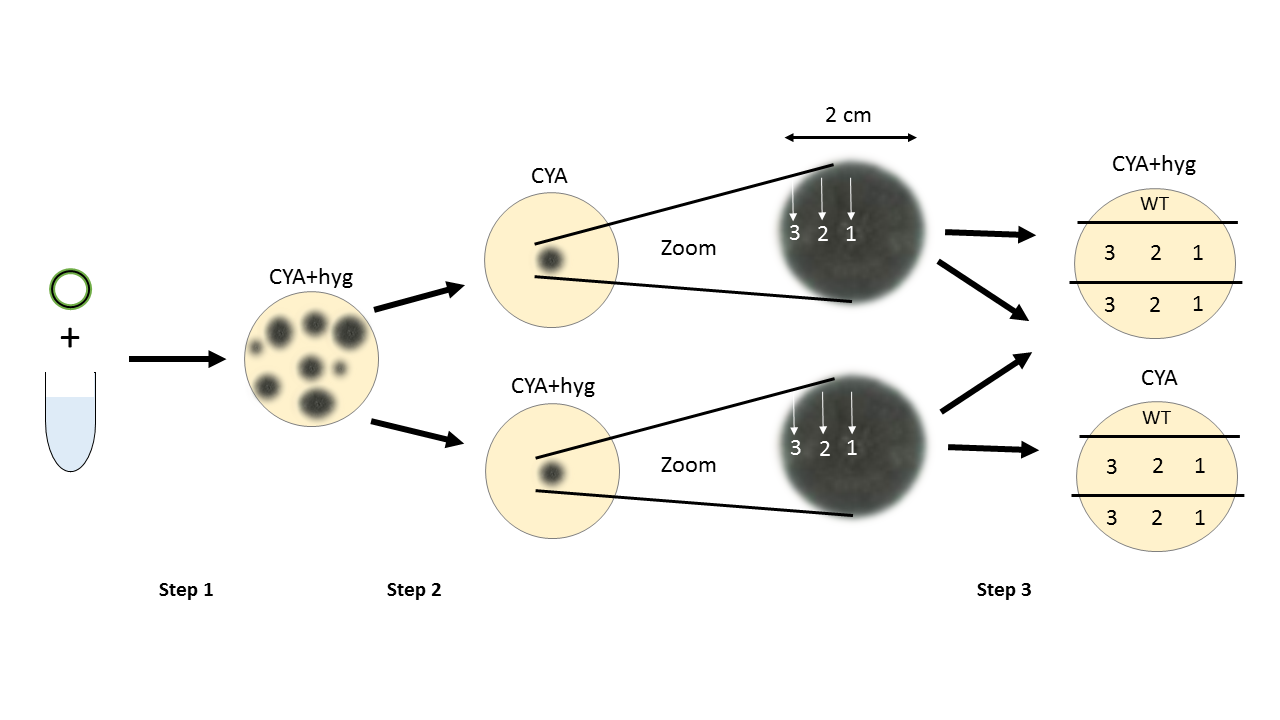


B


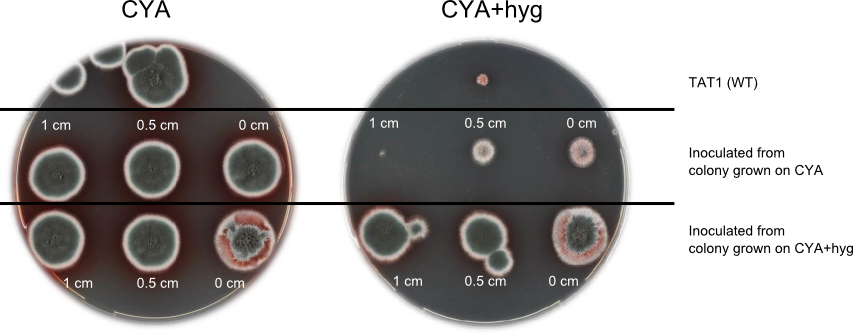

Supplement: S1 Fig — (DOCX) [file pone.0169712.s003.docx]
