# Supplementary material for: Genes Linked to Production of Secondary Metabolites in Talaromyces atroroseus Revealed Using CRISPR-Cas9
Source: PLoS One. 2017 Jan 5;12(1):e0169712. doi: 10.1371/journal.pone.0169712 (PMC5215926; doi:10.1371/journal.pone.0169712)

**S2 Fig. Deletion of the green pigment UA08_00425 (*albA*) in *T. atroroseus*. Second independent trial**. A-C) Plates resulting from co-transformation of pD-hyg-UA08_00425 and CRISPR-Cas9 vectors carrying three different protospacers, protospacer 1-3, respectively. D) *T. atroroseus* transformed with gene-targeting plasmid pD-hyg-UA08_00425 (pFC574) in the absence of a CRISPR-Cas9 vector.


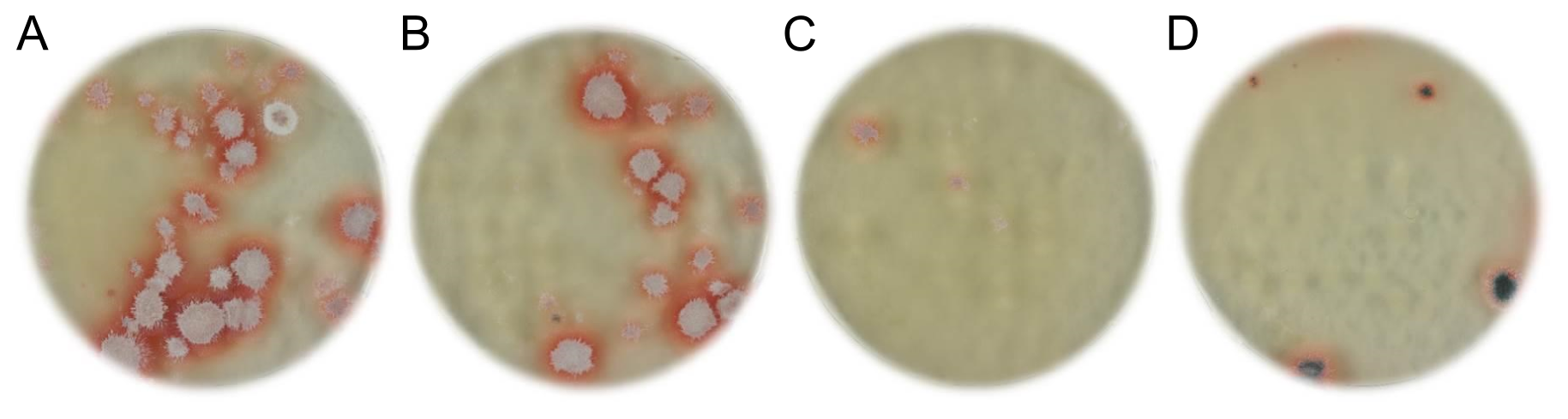

Supplement: S2 Fig — Second independent trial. (DOCX) [file pone.0169712.s004.docx]
