# Supplementary material for: Genes Linked to Production of Secondary Metabolites in Talaromyces atroroseus Revealed Using CRISPR-Cas9
Source: PLoS One. 2017 Jan 5;12(1):e0169712. doi: 10.1371/journal.pone.0169712 (PMC5215926; doi:10.1371/journal.pone.0169712)

**S3 Fig. Tissue PCR analysis for verification of *albA* deletion**.

The protocol for tissue PCR is described by Nødvig *et al*. For each PCR reaction, a dilution series of *T. atroroseus* mycelium was done to achieve optimal DNA template concentrations in one or more reactions. For each transformant, three PCR reactions were performed by adding approximately 1 mm^2^ mycelium template to three tubes by sequentially dipping the pipette tip with the biomass in each tube. Hence, the concentration of biomass was highest in the first tube and lower in the following two tubes. The resulting PCR reactions were loaded on an 1% agarose gel as sample 1, 2 and 3, respectively, as indicated on the gel picture. Lane labeled M includes the 1 kb ladder from New England Biolabs.

Three setups were performed for analysis of transformants to check for deletions of the green conidia pigment gene *albA* (UA08_00425). In the first PCR setup (A), the forward primer (ML581), which binds outside of the upstream *albA* targeting sequence, and the reverse primer (CSN105), which binds to the P*gpdA* promoter of the *hph* marker, are used for PCR. A successful gene replacement of *albA* with *hph* results in a PCR band of ~2.2 kb. Analyses of six randomly selected white transformants (corresponding gel lanes are indicated as colonies 1-6) using the first PCR setup are shown in (B) and (C). The number of transformants is indicated above each gel. (A) and (B) depict the same gel, but with different exposure times, hence allowing very weak bands to be visualized. Arrows point to successful PCR reactions.


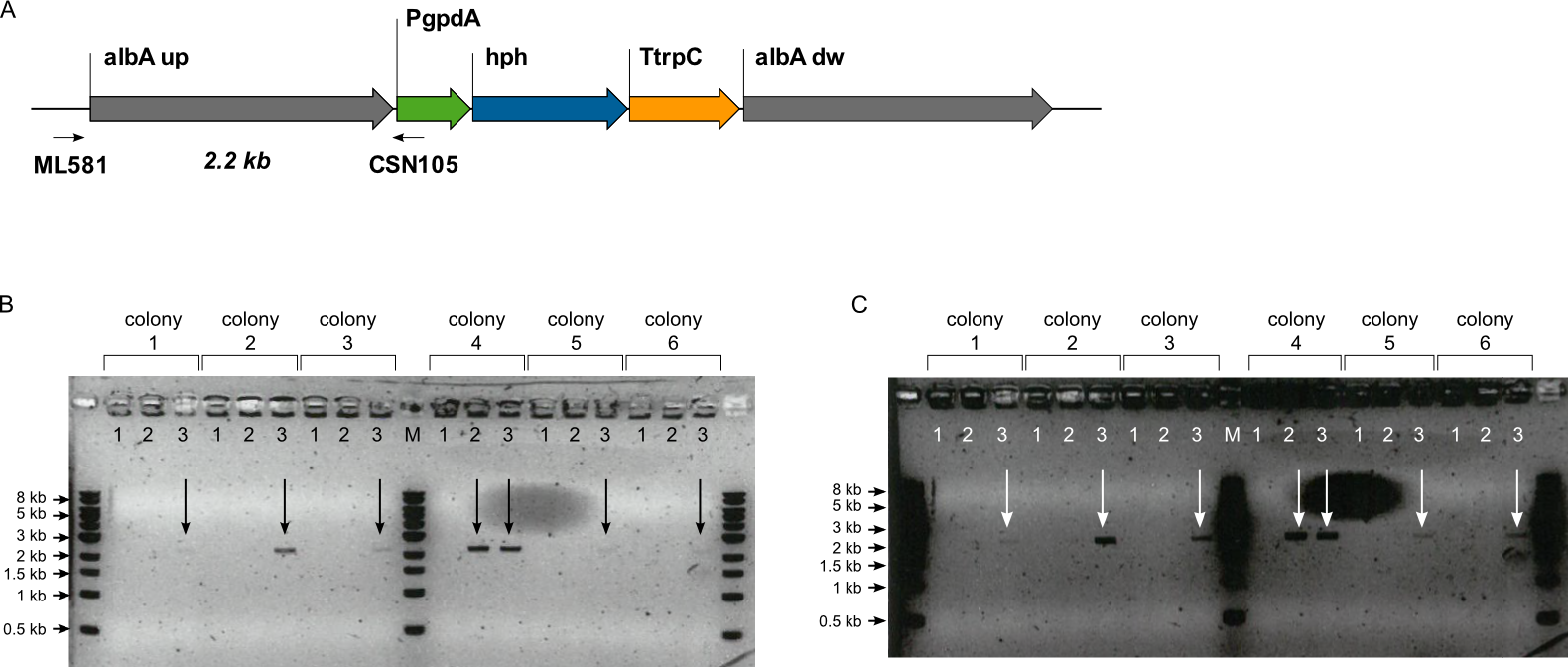


In the second PCR setup (D), the upstream primer (ML582) binds immediately upstream of the *albA* start codon, while the reverse primer (ML583) binds inside the *albA* ORF. The presence of a wild-type *albA* locus results in a band of ~0.65 kb. Analyses of six randomly selected white transformants using the second PCR setup are shown in (E) and (F). The number of transformants is indicated above each gel. (E) and (F) show the same gel, but with different exposure times. No visible bands can be detected indicating complete deletion of *albA*.


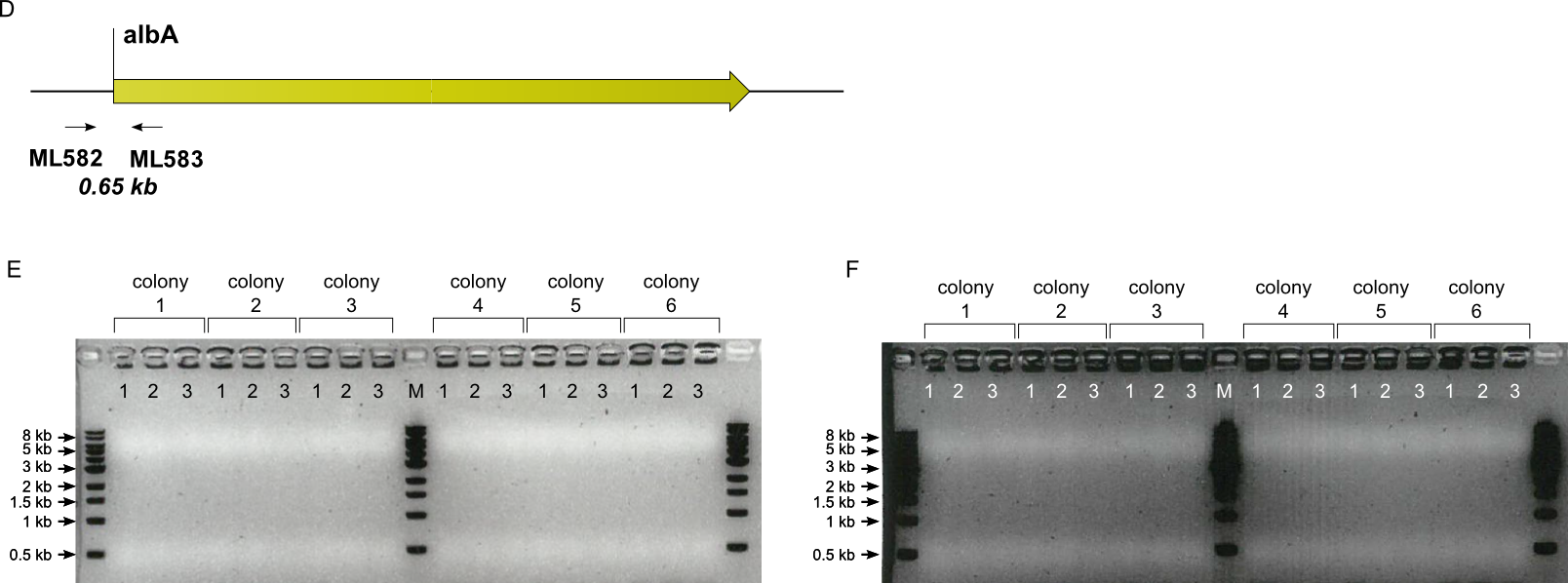


In the third PCR setup (G), the forward primer ML581 and the reverse primer B409 are used to amplify the *albA* locus. Both primers are situated outside the regions used as homology sequences of the *albA* gene-targeting substrate). The wild-type *albA* locus (upper cartoon) produces a band of 10.9 kb (upper cartoon) whereas a successful gene replacement of *albA* with *hph* (lower cartoon) results in a band of 6.6 kb. Analyses of a randomly selected white transformant and a wild-type strain (indicated as *albAΔ-1* and WT, respectively) using the third PCR setup are shown in (H). Arrows indicate the positions of the bands expected from wild-type *albA* (6.6 kb) and *albAΔ::hph* (10.7 kb). The position of an unknown unspecific PCR band of unknown origin is also indicated.


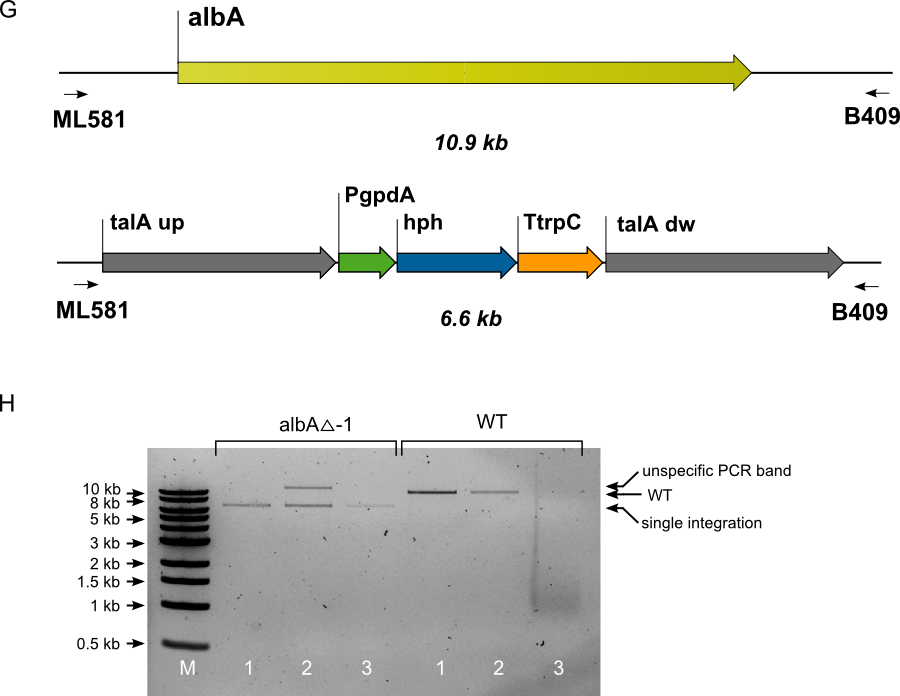

Supplement: S3 Fig — (DOCX) [file pone.0169712.s005.docx]
