# Supplementary material for: Genes Linked to Production of Secondary Metabolites in Talaromyces atroroseus Revealed Using CRISPR-Cas9
Source: PLoS One. 2017 Jan 5;12(1):e0169712. doi: 10.1371/journal.pone.0169712 (PMC5215926; doi:10.1371/journal.pone.0169712)

**S5 Fig. Tissue PCR analysis for verification of primer functionality**.

The protocol for tissue PCR is described by Nødvig *et al*. For each PCR reaction, a dilution series of *T. atroroseus* mycelium was done to achieve optimal DNA template concentrations in one or more reactions. For each transformant, three PCR reactions were performed by adding approximately 1 mm^2^ mycelium template to three tubes by sequentially dipping the pipette tip with the biomass in each tube. Hence, the concentration of biomass was highest in the first tube and lower in the following two tubes. The resulting PCR reactions were loaded on an 1% agarose gel as sample 1, 2 and 3, respectively, as indicated on the gel picture. A PCR reaction using purified genomic DNA as template was also included in the setup. Lane labeled M includes the 1 kb ladder from New England Biolabs.

The functionality of the primers used in S3 Fig (D-F) and S4 Fig (C-D) were tested using wild-type (WT) *T. atroroseus* tissue and gDNA. Using the primer sets for the *albA* locus (ML582+ML583) and the *talA* locus (ML515+ML516) results in a PCR band of ~0.65 kb in the WT strain in both loci. (A) shows positioning of primers at the *alba* and *talA* loci. (B) Agarose gel electrophoresis (1 %) of PCR samples obtained from WT *T. atroroseus* as well as from a single transformant of each of *albAΔ* and *talAΔ* are shown in (B).


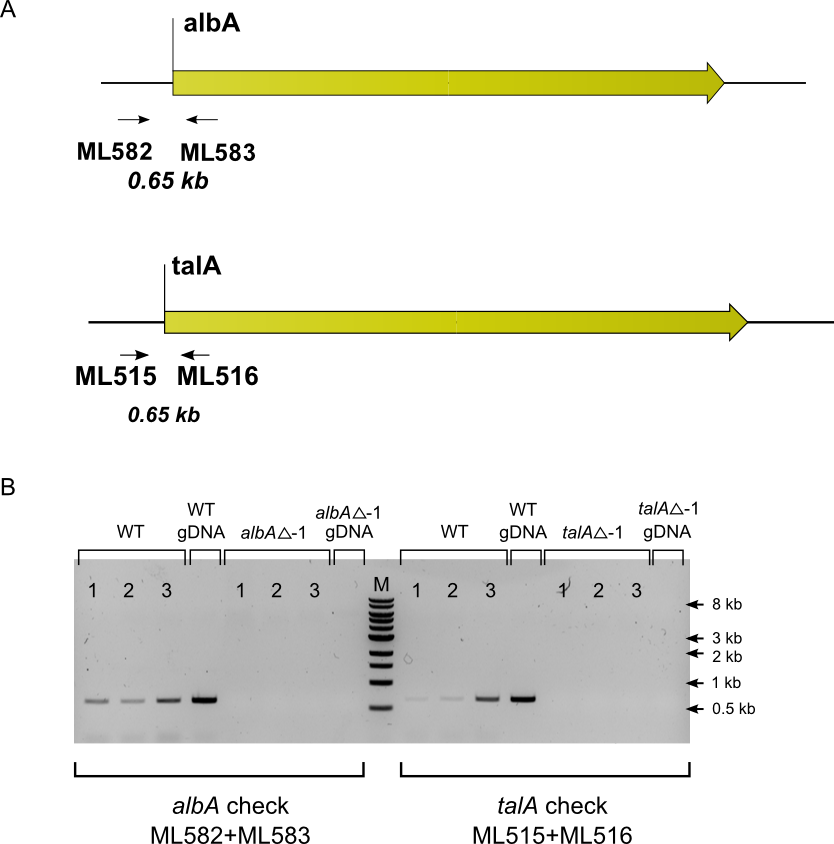

Supplement: S5 Fig — (DOCX) [file pone.0169712.s007.docx]
