# Supplementary material for: Genes Linked to Production of Secondary Metabolites in Talaromyces atroroseus Revealed Using CRISPR-Cas9
Source: PLoS One. 2017 Jan 5;12(1):e0169712. doi: 10.1371/journal.pone.0169712 (PMC5215926; doi:10.1371/journal.pone.0169712)
Supplement: S1 Table — (DOCX) [file pone.0169712.s008.docx]

**S1 Table. List of primers**

| UA08_00425-up-FU | ML571 | GGGTTTAAUTCACTACGTTCCGATTTTCGC | UA08_00425 deletion in T. atroroseus |
| --- | --- | --- | --- |
| UA08_00425-up-RU | ML572 | GGACTTAAUAACGGCTCTTTTTCTAATTGCTG | UA08_00425 deletion in T. atroroseus |
| UA08_00425-dw-FU | ML573 | GGCATTAAUCTCATTTTGGGTGTGTGGGAG | UA08_00425 deletion in T. atroroseus |
| UA08_00425-dw-RU | ML574 | GGTCTTAAUACTTATCGTCGTTTCTGATTGGAG | UA08_00425 deletion in T. atroroseus |
| gRNA-Tatro00425-1FU | ML559 | AGTAAGCUCGTCAACTTCCGACGGTCAGATGGGTTTTAGAGCTAGAAATAGCAAGTTAAA | UA08_00425 deletion in T. atroroseus (protospacer 3) |
| gRNA-Tatro00425-1RU | ML560 | AGCTTACUCGTTTCGTCCTCACGGACTCATCAGAACTTCCGGTGATGTCTGCTCAAGCG | UA08_00425 deletion in T. atroroseus (protospacer 3) |
| gRNA-Tatro00425-2FU | ML561 | AGTAAGCUCGTCAGCTCTTCAAGTACAAGAGGGTTTTAGAGCTAGAAATAGCAAGTTAAA | UA08_00425 deletion in T. atroroseus (protospacer 2) |
| gRNA-Tatro00425-2RU | ML562 | AGCTTACUCGTTTCGTCCTCACGGACTCATCAGAGCTCTCGGTGATGTCTGCTCAAGCG | UA08_00425 deletion in T. atroroseus (protospacer 2) |
| gRNA-Tatro00425-3FU | ML563 | AGTAAGCUCGTCGAGAAGTTCCCCCTGATCGGGTTTTAGAGCTAGAAATAGCAAGTTAAA | UA08_00425 deletion in T. atroroseus (protospacer 1) |
| gRNA-Tatro00425-3RU | ML564 | AGCTTACUCGTTTCGTCCTCACGGACTCATCAGGAGAAGCGGTGATGTCTGCTCAAGCG | UA08_00425 deletion in T. atroroseus (protospacer 1) |
| talA-up-FU | ML457 | GGGTTTAAUCTCACATTACTTTCATCTGGTCTCG | UA08_04451 deletion in T. atroroseus |
| talA-up-RU | ML458 | GGACTTAAUGACAGAATATATAAAATCAGAAAAGTCAAATAC | UA08_04451 deletion in T. atroroseus |
| talA-dw-FU | ML459 | GGCATTAAUTCGTAGATCGGGGCAGAGC | UA08_04451 deletion in T. atroroseus |
| talA-dw-RU | ML460 | GGTCTTAAUTGCATAGTTTGTCATTTTATCCCA | UA08_04451 deletion in T. atroroseus |
| gRNA-talA-FU | ML461 | AGTAAGCUCGTCAACTAAGCTAGAAGAACAGGGTTTTAGAGCTAGAAATAGCAAGTTAAA | UA08_04451 deletion in T. atroroseus |
| gRNA-talA-RU | ML462 | AGCTTACUCGTTTCGTCCTCACGGACTCATCAGAACTAACGGTGATGTCTGCTCAAGCG | UA08_04451 deletion in T. atroroseus |
| UA08_00425-upchk-F | ML581 | GACCAGTATCTTCGTATCTTTCGC | UA08_00425 deletion in T. atroroseus - check primer |
| UA08_00425-hkchk-F | ML582 | GATGCCTTGACATAATTTGTAACCA | UA08_00425 deletion in T. atroroseus - check primer |
| UA08_00425-hkchk-R | ML583 | CCTCCATCTCCATAGTGACTGGTA | UA08_00425 deletion in T. atroroseus - check primer |
| gpdA-p-int-rv | CSN105 | TTGGACGCCCTACAGATGC | Deletion up check primer (hph promoter) |
| talA-upchk-F | ML472 | ACCAACGAGCATAGTATCTCCGA | UA08_04451 deletion in T. atroroseus - check primer |
| talA-hkchk-F | ML515 | TGACCCTCTGGTGGTAGTTGG | UA08_04451 deletion in T. atroroseus - check primer |
| talA-hkchk-R | ML516 | GTCACCGCACATCAAGCCTAC | UA08_04451 deletion in T. atroroseus - check primer |
| TATR-albA-Dw-Chk-R | B409 | CTATTCTCGTACCGCATCCC | UA08_00425 deletion in T. atroroseus - check primer |
| TATR-talA-Dw-Chk-R | B410 | CCATTCCATGCAACACCTACGATC | UA08_04451 deletion in T. atroroseus - check primer |
