# Supplementary material for: Genes Linked to Production of Secondary Metabolites in Talaromyces atroroseus Revealed Using CRISPR-Cas9
Source: PLoS One. 2017 Jan 5;12(1):e0169712. doi: 10.1371/journal.pone.0169712 (PMC5215926; doi:10.1371/journal.pone.0169712)
Supplement: S2 Table — (DOCX) [file pone.0169712.s009.docx]

**S2 Table. List of plasmids**

| pFC330 | pCas9-pyrG | Empty CRISPR vector |
| --- | --- | --- |
| pFC332 | pCas9-hph | Empty CRISPR vector |
| pFC476 | pD-hyg | Deletion vector with *hph* marker |
| pFC574 | pAMA1-hph | AMA1 *hph* vector |
| pFC683 | pCas9-pyrG-talA | CRISPR plasmid for deletion of *talA* |
| pFC687 | pD-hyg-talA | *talA* deletion plasmid |
| pFC784 | pCas9-pyrG-UA08_00425-PS1 | CRISPR plasmid for deletion of *albA* |
| pFC785 | pCas9-pyrG-UA08_00425-PS2 | CRISPR plasmid for deletion of *albA* |
| pFC786 | pCas9-pyrG-UA08_00425-PS3 | CRISPR plasmid for deletion of *albA* |
| pFC789 | pD-hyg-UA08_00425 | *albA* deletion plasmid |
